# Multi-faceted roles of C1q/TNF-related proteins family in atherosclerosis

**DOI:** 10.3389/fimmu.2023.1253433

**Published:** 2023-10-13

**Authors:** Shuren Guo, Xiaohuan Mao, Jun Liu

**Affiliations:** ^1^ Department of Clinical Laboratory, Key Clinical Laboratory of Henan Province, The First Affiliated Hospital of Zhengzhou University, Zhengzhou, Henan, China; ^2^ Department of Clinical Laboratory, Henan Provincial People’s Hospital, People’s Hospital of Zhengzhou University, Zhengzhou, Henan, China; ^3^ College of Life Science and Technology, Xinjiang University, Xinjiang, China

**Keywords:** atherosclerosis, inflammation, metabolism, endothelial function, VSMCs

## Abstract

**Purpose of review:**

C1q/TNF-related proteins (CTRPs) are involved in the modulation of the development and prognosis of atherosclerosis (AS). Here, we summarizes the pathophysiological roles of individual members of the CTRP superfamily in the development of AS. Currently, there is no specific efficacious treatment for AS-related diseases, therefore it is urgent to develop novel therapeutic strategies aiming to target key molecules involved in AS.

**Recent findings:**

Recently, mounting studies verified the critical roles of the CTRP family, including CTRP1-7, CTRP9 and CTRP11-15, in the development and progression of AS by influencing inflammatory response, modulating glucose and lipid metabolism, regulating endothelial functions and the proliferation of vascular smooth muscle cells (VSMCs).

**Conclusions:**

CTRP family regulate different pathophysiology stages of AS. CTRP3, CTRP9, CTRP12, CTRP13 and CTRP15 play a clear protective role in AS, while CTRP5 and CTRP7 play a pro-atherosclerotic role in AS. The remarkable progress in our understanding of CTRPs’ role in AS will provide an attractive therapeutic target for AS.

## 1 Introduction

Atherosclerosis (AS) is the main pathological basis of coronary artery disease (CAD). AS is a complex and progressive disease involving inflammation, glucose and lipid metabolic disorder, endothelial dysfunction, proliferation and migration of vascular smooth muscle cells (VSMCs) (1). Atheroma was initiated by endothelial activation with recruitment of monocytes to the arterial intima (2), together with accumulation of lipids, adhesion of inflammatory cells to the arterial intima (3, 4). Lipid-loaded macrophages express scavenger receptors, taking up oxidized low-density lipoprotein (ox-LDL) particles and leading to foam cell formation (5, 6). VSMCs switch from “contractile” phenotype to a highly migratory and proliferative “synthetic” phenotype. Extracellular matrix synthesized by “synthetic” VSMCs forms a fibrous cap (7, 8).

The term C1q tumor necrosis factor-related protein (CTRP), originally introduced by Harvey Lodish and coworkers, describes a new family of secreted proteins highly conserved to adiponectin (9). The CTRP family contains 15 members. Recently, increasing evidences suggest that CTRP family plays a multiple role in inflammation regulation, glucose and lipid metabolism, endothelial functions. Thereby, CTRP family possesses a major influence on a variety of AS-related cells including endothelial cells dysfunction, the formation of foam cells and the proliferation of VSMCs. However, each CTRP displays varied alterations in the serum levels of atherosclerosis patients (**Table 1**) and exerts a unique influence on the progression of the disease (32).

**Table 1:** The correlation between CTRPs and atherosclerotic risk factors.

CTRPs	Variation of serum level in AS	BMI	Inflammatory factors	Reverse Cholesterol transport	Athersoclerosis (CMT, baPWV)	T2DM incident	CAD incident and severity	References
CTRP1	Increase		Positive	Inhibit	Positive	Positive	Positive	(10–15)
CTRP2	Increase	Positive		Promote			Positive	(16)
CTRP3	Decrease	Negative	Negative	Promote	Negative	Negative	Negative	(17, 18)
CTRP4	Increase						Positive	(19, 20)
CTRP4	Decrease						Negative	(21)
CTRP5	Increase				Positive			(14)
CTRP6	Increase	Positive		Promote		Positive		(22)
CTRP7	No related study	Positive	Positive	Promote		Positive		(23, 24)
CTRP9	Decrease			Promote	Positive			(25)
CTRP12	Decrease		Negative		Negative		Positive	(26–29)
CTRP13	Increase	Negative	Negative	Promote				(30)
CTRP15	Increase			Promote		Positive	Positive	(31)

Positive, Positive correlation between CTRP and atherosclerotic risk factors; Negative, negative correlation between CTRP and atherosclerotic risk factors; CMT, carotid intima-media thickness; baPWV, brachial ankle pulse wave velocity.

## 2 CTRPs as potential diagnostic and prognostic biomarker for AS

### 2.1 Markers with dual action on AS

CTRP1 was marked expressed in vascular wall tissue. Clinically, CTRP1 levels were higher in serum, endarterectomy specimens and aortic atherosclerotic plaques from CAD patients compared to controls (10–12). CTRP1 is positively correlated with interleukin-6 (IL-6), high-sensitivity C-reactive protein (hs-CRP) levels and the incidence of major adverse cardiovascular events (MACE) (13). CTRP2 is up-regulated in obesity and is positively correlated with body mass index (BMI) (16). CTRP2 over-expression improves insulin and lipid tolerance in diet-induced obese mice (33). Moreover, plasma triglyceride (TG) was significantly elevated in CTRP2-Knockout mice (16).

Previous studies showed contrary results on the association of serum CTRP4 levels and the CAD occurrence and severity. Gao J., et al. found increased serum CTRP4 levels were positively correlated with CAD occurrence and severity. CTRP4 combined with glycated hemoglobin has a better predictive value for CAD in type 2 diabetes mellitus (19). Dai, Y., et al. also demonstrated serum CTRP4 concentration was increased in patients with acute coronary syndrome (20). However, Liu, Z., et al. showed that serum CTRP4 were decreased in T2DM patients with Carotid atherosclerosis (CAS) compared to those without CAS, indicating that serum CTRP4 levels were negatively related to the risk of CAS in T2DM (21). Therefore, more clinical studies with large sample size are necessary to obtain more accurate results. The expression of CTRP6 in fat tissues was enhanced in obese and diabetic humans and mouse models (22).

### 2.2 Pro-atherosclerotic markers

CTRP1 and CTRP1/CTRP5 ratio were markedly higher in male AS patients with T2DM compared to controls, indicating that these CTRPs might have a causal role for cardio-metabolic risk in T2DM. In addition, the ratio of CTRP1 to CTRP5 in plasma is positively correlated with carotid intima-media thickness in the whole population (14). Lei, X. et al. found the positive association between elevated expression of CTRP2 and BMI in obesity (16). Ilbeigi, D., et al. demonstrated that serum levels of CTRP2 in CAD patients were independently associated with the progression of CAD, which indicates that CTRP2 might be considered as a novel biomarker for assessing the risk of CAD (34).

### 2.3 Anti-atherosclerotic markers

CTRP3 is a potent anti-inflammatory adipokine that inhibits pro-inflammatory pathways in monocytes and microcells during the development of CAD (32, 35, 36). Serum CTRP3 concentrations were significantly lower in CAD patients compared to controls. CTRP3 levels were significantly negatively correlated with glucose, BMI, smoking and hs-CRP levels, while positively related to HDL-C, adiponectin levels and CTRP3 gene expression adjusted for age and gender (17). Fadaei, R. et al. demonstrated that CTRP3 was significant independently negative associated with the presence of CAD (30). Moreover, Liu et al. and Wagner et al. found a difference in CTRP3 expression levels in male and female patients (26, 37). Hormonal status is speculated to underlie this sex-related difference. These results suggest that CTRP3 might be a new potential predictive biomarker in CAD (38).

CTRP9 was initially discovered as a well-known cardiovascular protective factor (39). CAD patients had a markedly lower serum CTRP9 level (25), indicating CTRP9 might be an independent protective factor of CAD. However, serum CTRP9 was higher in T2DM patients with AS by measuring brachial ankle pulse wave velocity (baPWV), suggesting that CTRP9 might be important in the regulation of arterial stiffness in humans (40). Several studies reported that CTRP12 levels were significantly lower in patients with CAD than those without CAD, and were independently associated with the risk of CAD (26–29). Liu Y et al. showed serum CTRP13 level was independently associated with HDL-C, insulin, HOMA-IR, HbA1c, TNF-α and BMI (30). The positive correlation between CTRP13 and HDL-C levels suggested a possible protective effect on lipid metabolism. Erbas IM, et al. also demonstrated that CTRP13 may serve as a novel biomarker for dyslipidemia in childhood obesity (41). On the contrary, Fadaei R et al. found that CTRP13 had negative correlation with pro-inflammatory cytokines such as TNF-α and IL-6, and it led to decreases in obesity and inflammation (30). In addition, higher serum levels of CTRP15 in CAD patients and the relation of CTRP15 with disease severity, pathogenic conditions such as insulin resistance and inflammation were demonstrated in previous study (31). These results suggest a possible compensatory response to the pathogenic conditions in CAD patients.

## 3 The mechanisms for the pleiotropic effects of CTRPs on AS

**Figure 1:**
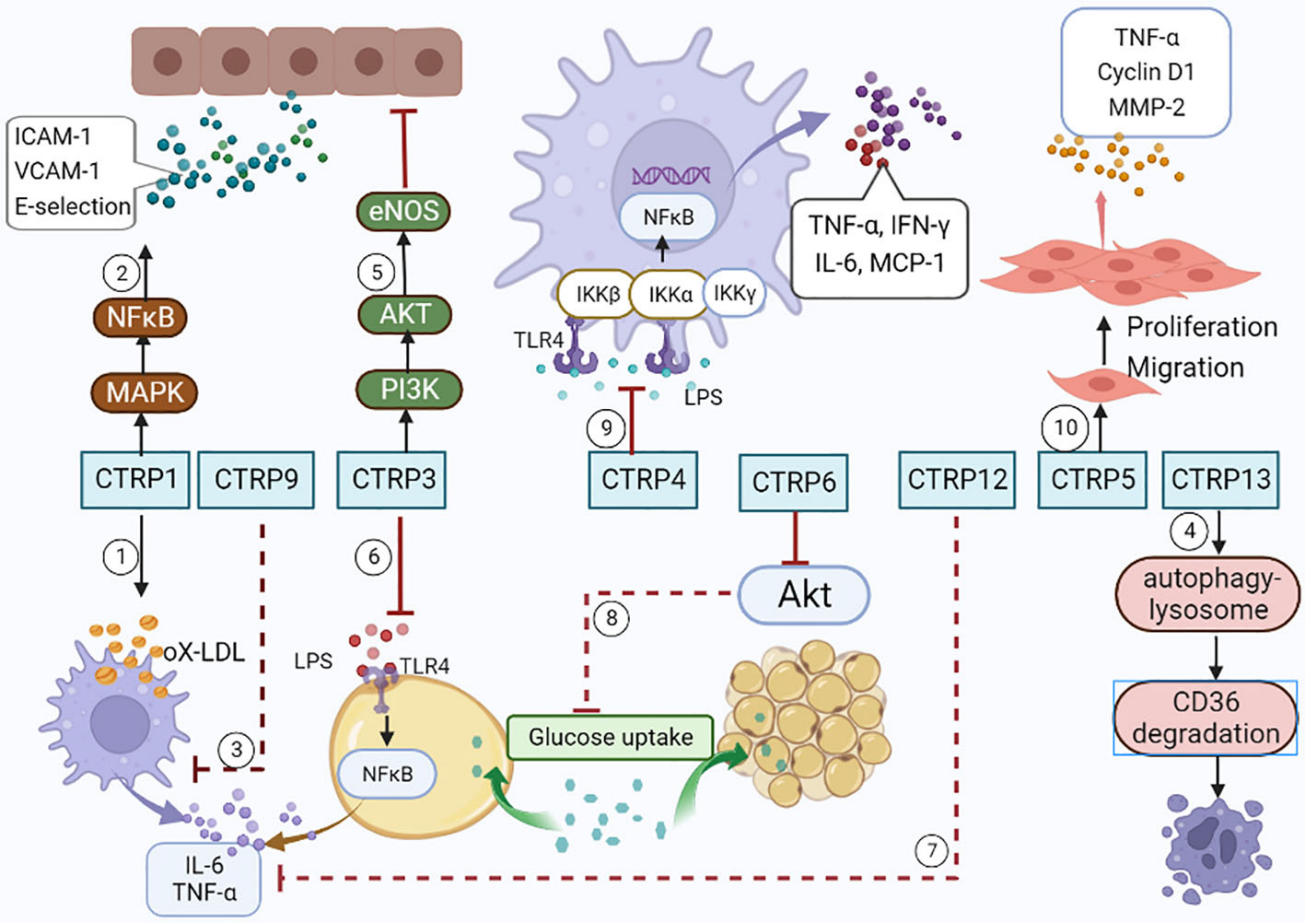
CTRPs and inflammation. ①CTRP1 increases the synthesis of inflammatory cytokines by activating MAPK/NF-kB signaling pathway. ②CTRP1 facilitates the secretion of inflammatory cytokines in macrophages stimulated with Ox-LDL. ③CTRP9 reduces the secretion of inflammatory cytokines in macrophages stimulated with Ox-LDL. ④CTRP13 accelerates macrophages autophagy through activating autophagy-lysosome pathways. ⑤CTRP3 inhibits endothelial inflammation by promoting PI3K/Akt/eNOS pathway. ⑥CTRP3 inhibits inflammatory properties in adipocyte cells by inhibiting the binding of LPS to toll-like receptor 4 (TLR4). ⑦CTRP12 reduces the expression of pro-inflammatory cytokines. ⑧CTRP6 reduces insulin-stimulated Akt phosphorylation and glucose uptake in adipocytes. ⑨CTRP4 alleviates the inflammatory cytokine storm by demoting of TLR4 internalization. ⑩CTRP5 facilitates the growth, migration, and inflammation of VSMCs by multiple pathways.

**Figure 2:**
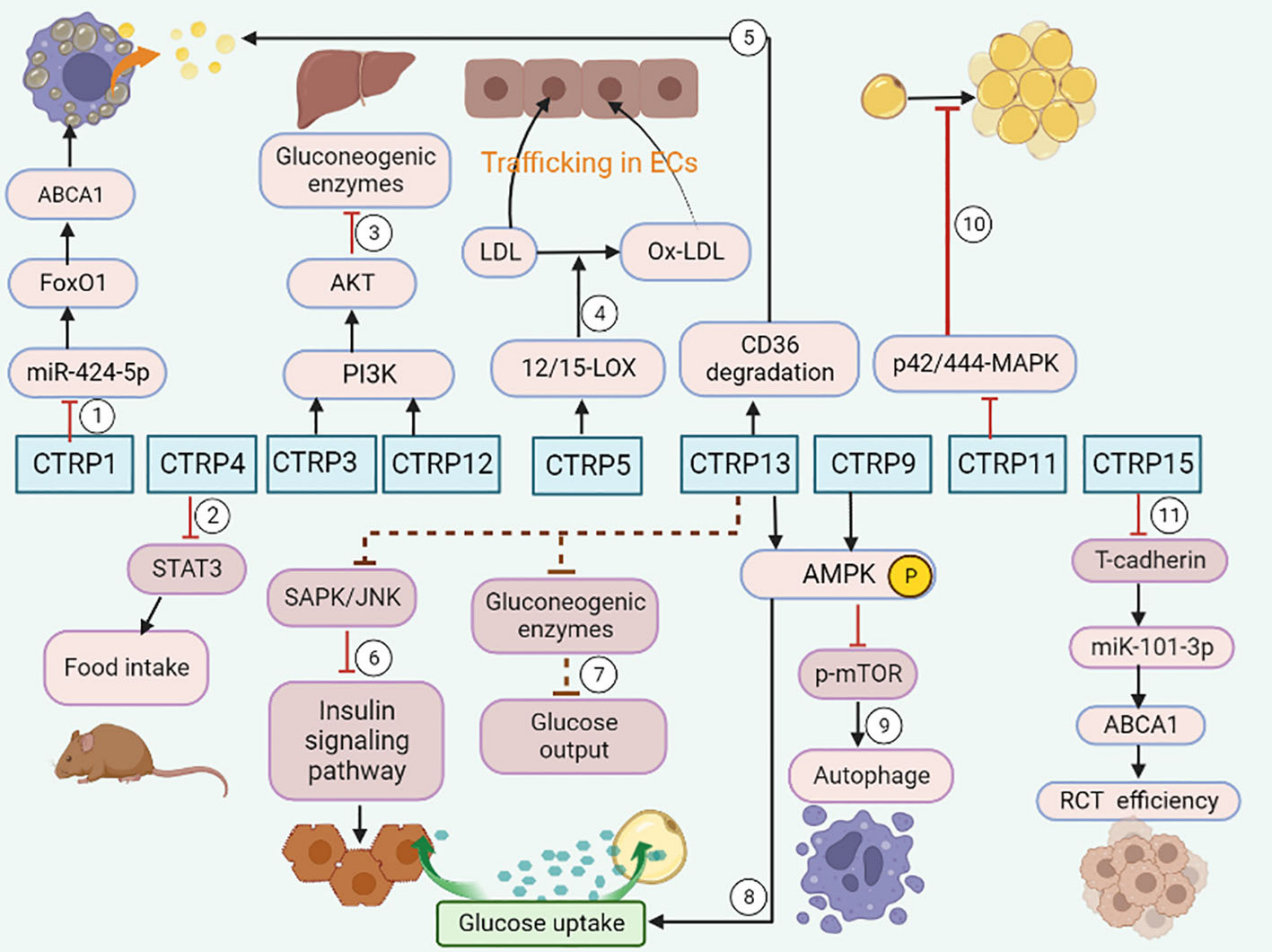
CTRPs and metabolism. ①CTRP1 promotes lipid accumulation through the miR-424-5p/FoxO1 pathway. ②CTRP4 suppresses food intake by inducing the activation of STAT3 signaling in mice. ③CTRP3 and CTRP 12 suppress gluconeogenesis by activating PI3K-Akt signaling pathway. ④CTRP5 promotes transcytosis and oxidation of LDL in endothelial cells via up-regulation of 12/15-LOX. ⑤CTRP13 increases cholesterol efflux in macrophage via autophagy-lysosome-dependent degradation of CD36. ⑥CTRP13 ameliorates insulin resistance in hepatocytes through suppression of the SAPK/JNK stress signaling. ⑦ CTRP13 reduces glucose output in hepatocytes by inhibiting the mRNA expression of gluconeogenic enzymes. ⑧CTRP13 stimulates glucose uptake in adipocytes, and hepatocytes via activation of the AMPK signaling pathway. ⑨CTRP9 promotes cholesterol efflux through AMPK/mTOR signaling pathway. ⑩Impaired adipogenesis is caused by a CTRP11-mediated decrease in p42/44-MAPK signaling. ⑪CTRP15 enhances RCT efficiency via the T-cadherin/miR-101-3p/ABCA1 pathway.

**Figure 3:**
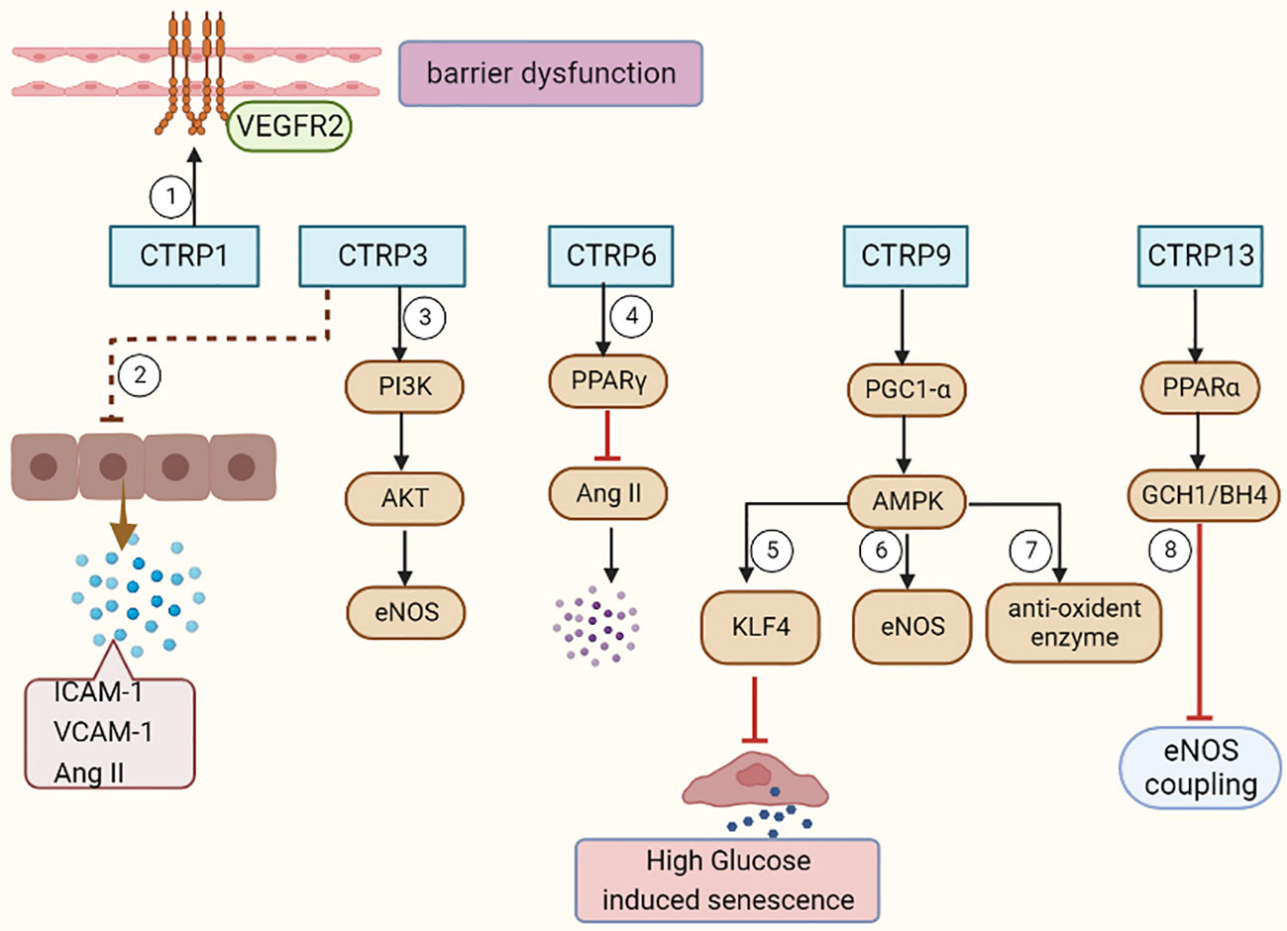
CTRPs and vascular endothelial functions. ①CTRP1 mediates vascular barrier dysfunction via activation of VEGFR2. ② CTRP3 decreases the Ang II, ICAM-1, and VCAM-1 expression in ECs. ③CTRP3 facilitates the activation of the PI3K/Akt/eNOS pathway in ECs. ④CTRP6 causes a significant decrease in AngII expression, further endothelial inflammation and apoptosis by improving PPARγ activation. ⑤CTRP9 inhibits endothelial cell senescence through the AMPKα/KLF4 signaling pathway. ⑥⑦CTRP9 reverses Ox-LDL-evoked decreases in antioxidant enzymes and eNOS in ECs. ⑧CTRP13 preserves endothelial function by regulating GCH1/BH4 axis-dependent eNOS coupling.

**Figure 4:**
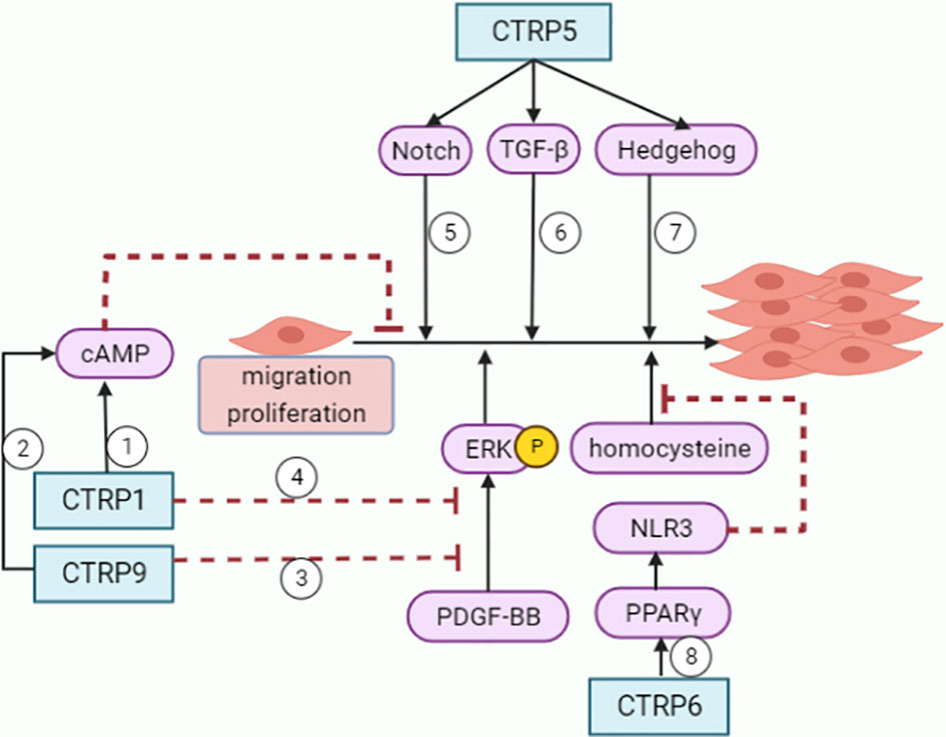
Roles of CTRPs in VSMCs. ①②CTRP1 and CTRP9 inhibit VSMCs growth through increasing cAMP levels. ③④CTRP1 and CTRP9 attenuate VSMCs proliferative activity and ERK phosphorylation in response to PDGF-BB. ⑤⑥⑦CTRP5 promotes inflammation, migration and proliferation in VSMCs with activation of Notch1, TGF-beta and hedgehog signaling pathways. ⑧CTRP6 inhibits homocysteine-induced proliferation and migration of VSMCs through PPARγ/NLRP3 pathway.

As an adiponectin paralog, CTRPs signals participate in a variety of pathophysiological processes. CTRP1-7, CTRP9 and CTRP11-15 can influence both the development and progression of AS by influencing inflammatory response (**Figure 1**), modulating glucose and lipid metabolism (**Figure 2**), regulating endothelial functions (**Figure 3**) and the proliferation of VSMCs (**Figure 4**).

### 3.1 The relationship of CTRPs with inflammation

AS is a chronic inflammatory disease of the arterial wall driven by innate and adaptive immune response (42, 43). Inflammation tunes each stage of the life cycle of atherosclerotic plaques (44). Atheroma initiation involves endothelial activation with recruitment of leucocytes to the arterial intima. VSMCs and infiltrating leucocytes can proliferate, but they also undergo various forms of cell death, leading to the formation of a lipid-rich ‘necrotic’ core. Inflammatory mediators participate in both the cell proliferate and cell death.

#### 3.1.1 CTRP1

Recombinant CTRP1 facilitated the secretion of IL-6, TNF-α, IL-1β, and monocyte chemoattractant protein-1 (MCP-1) in primary human macrophages stimulated with ox-LDL (45). CTRP1 also dramatically increased the mRNA levels of IL-6, ICAM-1, and MCP-1 in human aortic smooth muscle cells (hASMCs) (46). Lu et al. found that CTRP1 activated the p38 mitogen-activated protein kinase (MAPK)/nuclear factor (NF)-kB signaling pathway to promote the expression of adhesion molecules and synthesis of inflammatory cytokines (ICAM-1, VCAM-1 and E-selectin), leading to increased interaction between human peripheral blood monocytes and human ECs. These authors further demonstrated that loss of CTRP1 in apoE^-/-^ mice reduced atherosclerotic lesion area, along with a significant decrease in ICAM-1, VCAM-1, and E-selectin expression and macrophage infiltration within the plaques (10).

#### 3.1.2 CTRP3

CTRP3 expression was inhibited in ApoE^-/-^ mice compared to control mice. CTRP3 alleviates ox-LDL-induced inflammatory response by reducing pro-inflammatory factors CRP, TNF-α, IL-6, CD40, and CD40L in mouse aortic endothelial cells stimulated with ox-LDL. CTRP3 also inhibits ox-LDL induced endothelial inflammation by promoting phosphatidylinositol-3 kinase protein kinase B/endothelial nitric oxide synthase (PI3K/Akt/eNOS) pathway (47). Over-expression of CTRP3 elevated cell activity and decreased lactated hydrogenase release, accompanied by a marked reduction in cell apoptosis induced by ox-LDL (47). Furthermore, CTRP3 exhibited potent anti-inflammatory properties in adipocytes by inhibiting the binding of lipopolysaccharides (LPS) to toll-like receptor 4 (TLR4) (48).

#### 3.1.3 CTRP4

Adenovirus-mediated hypothalamic CTRP4 over-expression suppressed hypothalamic inflammation induced by high-fat diet in mice, which restored the impaired leptin signaling and decreased food intake (49). Additionally, CTRP4 over-expression alleviated the inflammatory cytokine storm by demoting of TLR4 internalization, which leading to NF-κB activation (50). Therefore, we can conclude that CTRP4 over-expression acts as an anti-inflammatory factor.

#### 3.1.4 CTRP5

CTRP5 exerts its pro-inflammatory effects by promoting the transport and oxidation of LDL by increasing 12/15-lipoxygenase (LOX) expression. CTRP5 facilitated the growth, migration, and inflammation of VSMCs through multiple pathways, leading to in-stent restenosis after coronary stent implantation (51).

#### 3.1.5 CTRP6

CTRP6-overexpressing mice or CTRP6-treated adipocytes had reduced insulin-stimulated Akt phosphorylation and glucose uptake. In addition, CTRP6 promoted a chronic state of low-level inflammation. On the contrary, CTRP6 deficiency reduced circulating inflammatory cytokines and pro-inflammatory macrophages in adipose tissue, while enhancing the activation of insulin-stimulated Akt in adipose tissue (22). Therefore, we speculated that CTRP6 was a novel metabolic/immune regulator linking obesity with adipose tissue inflammation and insulin resistance.

#### 3.1.6 CTRP9

CTRP9 could stabilize the mature plaques by reducing pro-inflammatory cytokines (IL-6, TNF-a, INF-γ and MCP-1) both in THP-1 macrophage foam cells (52) and the macrophages in the ApoE^-/-^ mice model (53). In addition, CTRP9 prevented adverse remodeling in the ischemic mouse heart by reducing MMP9 activation, which was associated with plaque vulnerability (54).

#### 3.1.7 CTRP12

Previous study showed CTRP12 could reduce the expression of pro-inflammatory cytokines and decrease macrophage accumulation within adipose tissue in obese mice. Clinical study reported that CTRP12 inhibited the secretion of inflammatory cytokines IL-6 and TNF-α in CAD patient (28, 55). These results indicate CTRP12 has anti-inflammatory and insulin sensitizing effects in the development and deterioration of CAD.

#### 3.1.8 CTRP13

CTRP13 inhibited the proliferation and migration of macrophages by down regulating lipid uptake, and then inhibited the plaque formation and AS development. Furthermore, CTRP13 delayed inflammatory responses during AS by promoting CD36-degradation through autophagy-lysosome pathways in macrophages, and thereby reduced the number of macrophages in lesions (56).

### 3.2 Effects of CTRPs on glucose and lipid metabolism

Glucose and lipid metabolism are the two major processes that increase the risk and severity of AS. Abnormal metabolism affects the activity of regulatory pathways, degree of inflammation, and the formation of coronary-plaque, thus contributing to the development of AS related disease (57). For instance, the formation of macrophage foam cells stimulated by ox-LDL is deemed an important cause of AS (58).

#### 3.2.1 CTRP1

Hyperglycemia is a well-known risk factor of AS. Plasma CTRP1 levels are higher in T2DM than controls in male. CTRP1 plays an important role in regulating body energy homeostasis and sensitivity to insulin, loss of CTRP1 disrupts glucose and lipid homeostasis (15). CTRP1 also improves glucose metabolism and insulin resistance in obese and STZ-induced diabetic mice. CTRP1 up regulates the protein level of leptin in blood, thermogenic gene expression in brown adipose tissue, and the gene expression responsible for lipolysis and glycolysis in white adipose tissue, thus reducing food intake and enhancing energy expenditure (59). Moreover, CTRP1 knockout mice fed a high-fat diet showed reduced liver and serum triglyceride and cholesterol levels due in part to increased hepatic AMP-activated protein kinase activation and decreased expression of lipid synthesis genes (15).

Recent study showed a novel mechanistic insight into its pro-atherosclerotic action. CTRP1 attenuated miR-424-5p levels and then augmented FoxO1 expression in the nucleus, which led to the reduced expression of ATP binding cassette transporter A1 (ABCA1). The primary function of ABCA1 is to mediate cholesterol efflux to apolipoprotein A-I (apoA-I) for generation of nascent high-density lipoprotein (HDL) particles. Briefly, CTRP1 decreased ABCA1 expression and promoted lipid accumulation through the miR-424-5p/FoxO1 pathway in THP-1 macrophage-derived foam cells (60).

#### 3.2.2 CTRP2

CTRP2, as the most similar to biological activities to those of adiponectin (61), is important in the regulation of whole body metabolism. Previous studies have revealed that mice over expressing CTRP2 exhibited improved insulin resistance and were better able to cope with acute lipid challenges than the control mice (33). On the contrary, Lei et al. found that the plasma TG and VLDL-TG in CTRP2 knockout mice were significantly elevated, and the absence of CTRP2 promoted hepatic TG secretion (16). Thus, we speculate that CTRP2 exerts its effects on the progression of AS through modulating glucose and lipid metabolism.

#### 3.2.3 CTRP3

Peterson et al. found that administration of recombinant CTRP3 to ob/ob mice could significantly reduce blood glucose levels by activating the Akt signaling pathway and inhibiting gluconeogenic enzymes in the liver (18).

#### 3.2.4 CTRP4

CTRP4 is a novel nutrient-responsive central regulator of food intake and energy balance (62). Serum CTRP4 levels are increased in leptin-deficient obese (ob/ob) mice. Central administration of recombinant CTRP4 inhibited food intake by inducing the activation of signal transducer and activator of transcription 3 (STAT3) signaling (63), and then altered the whole-body energy balance in both chow-fed and high-fat diet-fed mice. Serum CTRP4 concentrations decreased in patients with newly diagnosed T2DM (64), indicating CTRP4 is negatively associated with the risk of T2DM.

#### 3.2.5 CTRP5

CTRP5 activated signal transducer and activator of transcription 6 (STAT6) signaling, which in turn up-regulated the expression of 12/15-lipoxygenase (LOX). 12/15-LOX is a key enzyme which mediates LDL trafficking and oxidation. Genetic or pharmacological inhibition of 12/15-LOX dramatically reduced the deposition of ox-LDL in the sub-endothelial space and the development of AS. In short, CTRP5 is a novel pro-atherogenic cytokine, which promotes transcytosis and oxidation of LDL in endothelial cells via up regulating 12/15-LOX (65).

#### 3.2.6 CTRP7

In obese humans and Metabolic Syndrome (MetS) patients, circulating CTRP7 levels were significantly elevated and positively correlated with BMI, glucose, insulin, insulin resistance index, hemoglobin A1c, and triglyceride levels, which may be a novel biomarker related to metabolic diseases (23, 24). Expression of CTRP7 in liver was also significantly upregulated in obese humans, and was positively correlated with gluconeogenic genes. In mice, the expression of CTRP7 was differentially modulated in various tissues by fasting and refeeding, and by diet-induced obesity (66). Bioinformatics analysis revealed that CTRP7 was closely related to metabolism-related genes and signal pathways, further illustrating the association of CTRP7 with whole-body metabolism.

#### 3.2.7 CTRP9

In an AMPK/mTOR signaling pathway-dependent manner, CTRP9 promotes cholesterol efflux and inhibits foam cell formation by activating autophagy in ox-LDL-induced THP-1 macrophages (67).

#### 3.2.8 CTRP11

CTRP11 is mainly expressed in white and brown adipose, and its expression is acutely regulated by changes in metabolic state. Impaired adipogenesis was caused by a CTRP11-mediated decrease in p42/44-MAPK signaling and inhibition of mitotic clonal expansion. These results implicate that CTRP11 is a novel secreted regulator of adipogenesis (68). Interestingly, CTRP11 deficiency affects metabolic parameters in a sexually dimorphic manner. Significantly higher fasting serum ketones and reduced physical activity were only found in Ctrp11-KO female mice, which can be reversed by refeeding (69). Although it is unclear whether sex hormones directly modulate CTRP expression levels, these sexually dimorphic patterns are observed in several other CTRP family members, such as CTRP5, CRTP9 (70), CTRP11, CTRP13 (71) and adiponectin.

#### 3.2.9 CTRP12

In apoE^-/-^ mice fed a Western diet, CTRP12 reduced the area of atherosclerotic lesion by increasing the plasma level of HDL-C, promoting reverse cholesterol transport (RCT) and alleviating inflammatory response (29). CTRP12 also directly activated the PI3K-Akt signaling pathway to inhibit gluconeogenesis and promote glucose uptake in the obese and diabetic mouse (72).

#### 3.2.10 CTRP13

CTRP13 is a secreted adipokine that can ameliorate abnormal glucose and lipid metabolism (56). CTRP13 has been verified to stimulate glucose uptake in adipocytes, myotubes, and hepatocytes in vitro by activating the AMPK signaling pathway. CTRP13 diminishes lipid-induced insulin resistance in hepatocytes through inhibiting the SAPK/JNK stress signaling that damages the insulin signaling pathway. In addition, CTRP13 reduces glucose output in hepatocytes by inhibiting the mRNA expression of gluconeogenic enzymes, glucose-6-phosphatase and the cytosolic form of phosphoenolpyruvate carboxykinase. Taken together, these results indicate that CTRP13 plays an important role in glucose homeostasis (71).

Previous studies showed that upregulation of CD36 inhibited cholesterol efflux through the activation of PKCθ (73). Additionally, CTRP13 inhibited AS via autophagy- lysosome-dependent degradation of CD36, leading to the increase of cholesterol efflux in macrophage (56). Furthermore, CTRP13 hydrolyzed cholesterol droplets stored in macrophages, which attenuates cholesterol influx and promotes reverse cholesterol transport, thus inhibiting the formation of foam cells by decreasing the uptake of Ox-LDL (56) and the progression of AS (74, 75).

#### 3.2.11 CTRP15

CTRP15 over-expression significantly decreased atherosclerotic plaque lesions through increasing reverse cholesterol transport (RCT) efficiency and circulating HDL-C levels in ApoE^-/-^ mice. Consistently, in vitro, over-expression of CTRP15 inhibited intracellular lipid accumulation and promoted cholesterol efflux from macrophages (76). Mechanism study verified that CTRP15 enhanced RCT efficiency and increased plasma HDL-C levels via the T-cadherin/miR-101-3p/ABCA1 pathway. Targeting CTRP15 may serve as a novel and promising therapeutic strategy for atherosclerotic diseases.

### 3.3 Roles of CTRPs in regulating vascular endothelial functions

Endothelial cell dysfunction, as a hallmark of AS, is characterized by decreased bioavailability of nitric oxide (NO), increased production of reactive oxygen species (ROS), impaired vasodilation and decreased angiogenesis potential (77). Ox-LDL accumulation is one of the critical determinants in endothelial dysfunction. The endothelial apoptosis in response to ox-LDL promotes the lipids deposition, foam cell formation, and the development of atherosclerotic plaque (78, 79).

#### 3.3.1 CTRP1

Endothelial hyper-permeability is a main determinant factor that contributes to the accelerated development of atherosclerotic lesions at hemodynamically disturbed sites. CTRP1 expression was significantly elevated in vascular endothelial cells under disturbed flow compared to steady laminar flow in mouse aorta (80). The activation of vascular endothelial growth factor receptor 2 (VEGFR2) by CTRP1 might be related to vascular hyper-permeability. CTRP1 is a mechanically sensitive pro-inflammatory factor that mediates disturbed flow-induced vascular barrier dysfunction. Inhibition of CTRP1 may inhibit the pathogenesis of AS at early stage.

#### 3.3.2 CTRP3, CTRP5 and CTRP6

Over-expressed CTRP3 caused a decrease in Angiotensin II (AngII), ICAM-1, and VCAM-1 expression, which regulated the balance between ET-1 and NO. Incremental CTRP3 increased the expression of p-PI3K, p-Akt and p-eNOS, indicating that CTRP3 facilitated the activation of PI3K/Akt/eNOS pathway (47). CTRP3 ameliorated uric acid-induced endothelial inflammation and oxidative stress, possibly by inhibiting TLR4-mediated inflammation and down-regulating oxidative stress (81). Globular form CTRP5 is a novel molecule that leads to vascular EC dysfunction through Nox1-mediated mitochondrial apoptosis in diabetes, which indicates that interventions blocking gCTRP5 may protect diabetic EC function (82). AngII has been regarded as a major contributor to the incidence of vascular endothelial dysfunction (83). Over-expression of CTRP6 improved peroxisome proliferator-activated receptor gamma (PPARγ) activation, which caused a significant decrease in AngII expression, and vascular endothelial inflammation and apoptosis (83). On the contrary, silencing CTRP6 inhibited PPARγ activation and exacerbated AngII-mediated vascular endothelial dysfunction and apoptosis.

#### 3.3.3 CTRP9

CTRP9 exerts a significant protective role in endothelial cells. CTRP9 attenuates palmitic acid-induced endothelial cell senescence via increasing autophagy (84). Sun, H et al. found that CTRP9 treatment reversed ox-LDL-evoked decreases in antioxidant enzymes as well as eNOS. CTRP9 ameliorates ox-LDL-induced endothelial dysfunction via activation of proliferator-activated receptor γ co-activator 1α (PGC1-α)/adenosine monophosphate-activated protein kinase (AMPK)-mediated antioxidant enzyme induction (85). CTRP9 also exerts vasculoprotective effects via the adiponectin receptor 1/AMPK/eNOS dependent/NO mediated signaling pathway (86). Moreover, CTRP9 might protect endothelial oxidative damage via AdipoR1-SIRT1-PGC1-alpha signaling pathway (87) and inhibit endothelial cell senescence through the AMPKα/KLF4 signaling pathway under high glucose (88). The endothelial cells generate more ROS production under a high glucose environment, along with decreased mitochondrial biogenesis. In contrary, the treatment of CTPR9 significantly increased the activity of cytochrome c oxidase, indicating an induction of mitochondrial biogenesis (87).

#### 3.3.4 CTRP13

Previous study showed CTRP13 supplement rescued the impaired endothelium-dependent relaxation ex vivo in the db/db mouse aortae and in high glucose-treated mouse aortae. CTRP13 preserves endothelial function in diabetic mice by increasing GTP cyclohydrolase 1 (GCH1) expression and tetrahydrobiopterin (BH4) levels to ameliorate eNOS coupling (89). More importantly, CTRP13 rescued high glucose-induced inhibition of protein kinase A (PKA) activity. GCH1 transcription was activated by the phosphorylation and recruitment of PPARα, thus improved the endothelial relaxation. Together, these results suggested that CTRP13 preserves endothelial function in diabetic mice by regulating GCH1/BH4 axis-dependent eNOS coupling.

#### 3.3.5 CTRP14

CTRP14 is synthesized and secreted mainly by the brain and adipose tissues. The globular domain of C1ql1/Ctrp14 and C1ql4/Ctrp11 proteins directly stimulate the angiogenesis of endothelial cells activation of ERK1/2 signal pathway (90). However, Guan et al. illustrated that CTRP14 was largely dispensable for AS formation in ApoE-deficient (apoE^-/-^) mice and does not improve atherosclerotic plaque formation in the aorta (91).

### 3.4 Roles of CTRPs in VSMCs migration and proliferation

Accumulation of VSMCs is an important event in atherogenesis (92). VSMCs go through a phenotypic switching in AS. Under basal conditions, VSMCs are in the quiescent stage, which is less proliferative and has a relatively low turnover rate (93). Upon vascular injury, the contractile VSMCs switch to synthetic phenotype and undergo proliferation, as well as migration from vascular media to the injury site, to propagate wound repairing (93). VSMCs may also adopt to other phenotypes, including foam cells within atherosclerotic plaques that masquerade as macrophages (94).

CTRP1 and CTRP9 prevent neointima formation by inhibiting VSMCs growth through cyclic AMP (cAMP) -dependent pathway (95, 96). Treatment of VSMCs with CTRP1 or CTRP9 protein attenuated proliferative activity and ERK phosphorylation in response to platelet-derived growth factor-BB (PDGF-BB). CTRP1 or CTRP9 treatment also can increase cAMP levels. Furthermore, compared to control WT mice, CTRP1-knockout mice showed increased neointimal thickening and increased numbers of proliferating cells in neointima following injury (95).

CTRP5 promoted inflammation, migration and proliferation in hASMCs in wound-healing (51). CTRP5 activated Notch1, TGF-β and hedgehog signaling pathways, thus concentration-dependently induced the expression of MMP-2, cyclin D1 and TNF-α in hASMCs.

CTRP6 inhibits VSMCs proliferation and migration induced by PDGF-BB (97). Besides, CTRP6 also inhibited homocysteine induced proliferation, migration, and dedifferentiation of VSMCs through PPARγ/NLRP3 pathway (98).

## 4 Conclusion and future directions

At present, enormous evidence has shown that CTRPs are closely related to the risk factors of AS, such as obesity, hyperlipidemia, hyperglycemia, inflammation. The level of CTRPs in serum is expected to serve as a new type biomarker for AS, which can be combined with other biomarkers to evaluate and predict the occurrence and development of AS.

CTRPs influence vascular biology and atherosclerosis through various highly specialized functions that regulate and coordinate inflammatory response, glucose and lipid metabolism, endothelial functions and the proliferation of VSMCs (**Supplementary Table 1**). Firstly, CTRP1 increases the synthesis and facilitates the secretion of inflammatory cytokines in macrophages. CTRP5 facilitates the growth, migration, and inflammation of VSMCs. In contrast, CTRP3 inhibits inflammation in endothelial cells and adipocytes. CTRP6 relieves endothelial inflammation and apoptosis by improving PPAR-γ activation. CTRP4, CTRP9 and CTRP12 reduce the secretion of inflammatory cytokines in macrophages. CTRP13 accelerates macrophages autophagy. Secondly, CTRP family ameliorates abnormal glucose and lipid metabolism in a various ways and mechanisms. In vitro studies showed that CTRP9 and CTRP13 increase cholesterol efflux in macrophages. Adipogenesis is impaired by a CTRP11-mediated decrease in p42/44-MAPK signaling. CTRP3 and CTRP12 suppress gluconeogenesis in hepatocytes. CTRP13 ameliorates insulin resistance and reduces glucose output in hepatocytes. In addition, CTRP13 stimulates glucose uptake in adipocytes and hepatocytes. In vivo studies also demonstrated that CTRP4 suppresses food intake in mice. On the contrary, some other CTRPs were reported to accelerate AS by modulating glucose and lipid metabolisms. For example, CTRP1 promotes lipid accumulation in macrophages. CTRP5 promotes transcytosis and oxidation of LDL in endothelial cells. CTRP6 reduces glucose uptake in adipocytes. Thirdly, although CTRP1 mediates vascular barrier dysfunction via activation of VEGFR2, most CTRPs have been confirmed to exert protective roles for endothelial cells. CTRP3 facilitates the activation of the PI3K/Akt/eNOS pathway in ECs. CTRP9 reverses Ox-LDL-evoked decreases in antioxidant enzymes and eNOS in ECs, further inhibits endothelial cell senescence. CTRP13 preserves endothelial function by regulating GCH1/BH4 axis-dependent eNOS coupling. Fourthly, CTRP1 and CTRP9 attenuate VSMCs proliferative activity in response to PDGF-BB. Furthermore, CTRP6 inhibits homocysteine-induced proliferation and migration of VSMCs through PPARγ/NLRP3 pathway. Nevertheless, CTRP5 promotes inflammation, migration and proliferation in VSMCs with activation of Notch1, TGF-beta and hedgehog signaling pathways.

Since part of the CTRPs play a complex dual regulatory roles in AS, and most of the current studies focus on the role of CTRPs in cells in vitro, animal experiments are relatively few, it is difficult to comprehensively evaluate whether a single CTRP plays a pro-atherosclerotic or anti-atherosclerotic role in the progression of AS in human. But in vitro researches demonstrated that some CTRPs such as CTRP3, CTRP9, CTRP12, CTRP13 and CTRP15, play a clear protective role in AS, while CTRP5 and CTRP7 play a pro-atherogenic role in AS. Advances in the understanding of CTRPs biology and their translation into therapeutic agents to reduce the risk of AS are great needed. The remarkable progress in our understanding of CTRPs’ role in AS will provide an attractive therapeutic target for AS.

## Author contributions

SG: Writing – review & editing. XM: Writing – original draft. JL: Writing – original draft.

## Supplementary material

The Supplementary Material for this article is correctly linked to https://www.frontiersin.org/articles/10.3389/fimmu.2023.1253433/full#supplementary-material
