# Supplementary material for: Multi-faceted roles of C1q/TNF-related proteins family in atherosclerosis
Source: Front Immunol. 2023 Oct 13;14:1253433. doi: 10.3389/fimmu.2023.1253433 (PMC10611500; doi:10.3389/fimmu.2023.1253433)
Supplement: Supplementary file 1 [file Table_1.docx]

| Supplement Table 1 Roles of CTRP family associated with Atherosclerosis in multiple ways | | | | | | | |  |
| --- | --- | --- | --- | --- | --- | --- | --- | --- |
| Members of CTRPs | Disease models |  | promotive or inhibitory roles |  | mechanisms | references |  |  |
| CTRP1 | ApoE^-/-^ mice, CTRP^-/-^ mice, ApoE^-/-^ CTRP^-/-^ mice, human macrophages, human aortic smooth muscle cells |  | Promotive |  | CTRP1 increases the expression of inflammatory cytokines, adhesion molecules and chemokines, and promotes the formation of macrophages in atherosclerotic plaques. | 10 | | |
|  | THP-1 macrophage-derived foam cells |  | Promotive |  | CTRP1 decreases ABCA1 expression and promotes lipid accumulation through the miR-424-5p/FoxO1 pathway. | 60 | | |
|  | CTRP1 transgenic mice |  | Inhibitory |  | CTRP1 enhances fatty acid oxidation and improves insulin sensitivity. |  | | |
|  | obese and STZ-induced diabetic mice |  | Inhibitory |  | CTRP1 improves glucose metabolism and insulin resistance. | 59 | | |
|  | CTRP1 knock out mice fed a high-fat diet |  | Inhibitory |  | CTRP1 reduces liver and serum triglyceride and cholesterol levels by increasing hepatic AMPK activation and reducing the expression of lipid synthesis genes. | 15 | | |
|  | Mice subjected to wire-induced injury of left femoral arteries |  | Inhibitory |  | CTRP1 inhibits the proliferation of human VSMCs and ameliorates neointimal hyperplasia in response to PDGF-BB through the cAMP- dependent pathway. | 95 | | |
|  | mouse aorta |  | Promotive |  | Activation of Vascular Endothelial Growth Factor Receptor 2 (VEGFR2) by CTRP1 might be involved in vascular hyper-permeability under disturbed flow condition. | 80 | | |
| CTRP2 | mice over expressing CTRP2, control mice |  | Inhibitory |  | CTRP2 improved insulin resistance. | 33 | | |
|  | CTRP2 knockout mice |  | Promotive |  | CTRP2 elevates plasma TG by promoting hepatic TG secretion. | 19 | | |
|  |  |  |  |  |  |  | | |
| CTRP3 | ob/ob mice |  | Inhibitory |  | CTRP3 lowered blood glucose levels by activation of the Akt signaling pathway and suppression of gluconeogenic enzymes in the liver. | 18 | | |
|  | Endothelial Cells in ApoE-/- mice |  | Inhibitory |  | CTRP3 alleviates ox-LDL-induced inflammatory response and endothelial dysfunction by activating the pi3k/akt/enos pathway. | 39 | | |
| CTRP5 | Human aortic endothelial cells |  | Promotive |  | CTRP5 promotes transcytosis of LDL across endothelial monolayers, and as well as the oxidative modification of LDL in endothelial cells. | 65 | | |
|  | endothelial cells |  | Promotive |  | up-regulation of 12/15-lipoxygenase |  | | |
|  | VSMCs of CAD patients with in-stent restenosis after coronary stent implantation |  | Promotive |  | CTRP5 facilitates the growth, migration, and inflammation of VSMCs by multiple pathways. | 51 | | |
|  | diabetic vascular EC |  | Promotive |  | Globular form CTRP5 contributes to diabetic vascular EC dysfunction through Nox1-mediated mitochondrial apoptosis. | 82 | | |
| CTRP6 | adipose tissue |  | Promotive |  | CTRP6 reduces insulin-stimulated Akt phosphorylation and glucose uptake, and promotes a chronic state of low-grade inflammation. | 22 | | |
|  | VSMCs |  | Inhibitory |  | CTRP6 inhibits PDGF-BB-induced VSMCs proliferation and migration. | 96 | | |
|  | Endothelial cells |  | Inhibitory |  | CTRP6 improves peroxisome proliferator-activated receptor gamma (PPARγ) activation and alleviates AngII-induced vascular endothelial dysfunction. | 83 | | |
| CTRP7 | liver and fat of db/db, and ob/ob mice |  | Promotive |  | CTRP7 overexpression facilitated oxidative stress and suppressed the phosphorylation of insulin signaling molecules in hepatocytes. | 24 | | |
| CTRP9 | Human THP-1 monocytes |  | Inhibitory |  | CTRP9 promotes cholesterol efflux and inhibits foam cell formation by activating autophagy. | 67 | | |
|  | ApoE^-/-^ mice |  | Inhibitory |  | CTRP9 stabilizes the mature plaques by reducing pro-inflammatory cytokines in macrophages. | 53 | | |
|  | Ox-LDL-induced endothelial |  | Inhibitory |  | CTRP9 ameliorates Ox-LDL-induced endothelial dysfunction via PGC-1α/AMPK-mediated antioxidant enzyme induction and the adiponectin receptor 1/AMPK/eNOS dependent/NO mediated signaling pathway. | 85 | | |
|  | high glucose-induced endothelial cells |  | Inhibitory |  | CTRP9 induces mitochondrial biogenesis and protects high glucose- induced endothelial oxidative damage via AdipoR1-SIRT1 -PGC-1alpha signaling pathway.  CTRP9 inhibites the high glucose-induced endothelial cell senescence through the AMPKα/KLF4 signialing pathway. | 87  88 | | |
|  | VSMCs |  | Inhibitory |  | CTRP9 inhibits VSMCs growth via cAMP-dependent mechanism. | 98 | | |
| CTRP12 | Leptin-deficient ob/ob mice, and diet-induced obese mice |  | Inhibitory |  | CTRP12 improves insulin sensitivity, lowers blood glucose, suppresses gluconeogenesis and promotes glucose uptake. | 72 | | |
|  | apoE^-/-^ mice fed a western diet |  | Inhibitory |  | CTRP12 promots reverse cholesterol transport (RCT), and alleviates inflammatory response | 29 | | |
|  | CAD patients |  | Inhibitory |  | CTRP12 could reduce the expression and the secretion of pro-inflammatory cytokines, and decrease macrophage accumulation. | 28 | | |
| CTRP13 | Primary peritoneal macrophages |  | Inhibitory |  | CTRP13 suppresses CD36 levels, ox-LDL uptake and foam-cell formation, and promotes cholesterol outflow. | 56 | | |
|  | hepatocytes |  | Inhibitory |  | CTRP13 ameliorates lipid-induced insulin resistance through suppression of the SAPK/JNK stress signaling. | 71 | | |
|  | db/db mouse aortae and high glucose-treated mouse aortae |  | Inhibitory |  | CTRP13 preserves endothelial function in diabetic mice by regulating GCH1/BH4 axis-dependent eNOS coupling and inhibiting protein kinase A (PKA) activity. | 89 | | |
| CTRP14 | endothelial cells |  | Promotive |  | The globular domain of C1ql1/Ctrp14 and C1ql4/Ctrp11 proteins directly stimulate the angiogenesis of endothelial cells activation of ERK1/2 signal pathway. | 90 | | |
|  | ApoE^-/-^ mice |  | No significant role |  | C1QL1/CTRP14 is largely dispensable for atherosclerosis formation in ApoE-deficient mice and does not improve atherosclerotic plaque formation in the aorta. | 91 | | |
| CTRP15 | ApoE^-/-^ mice and macrophages |  | Inhibitory |  | CTRP15 enhances RCT efficiency and increases plasma HDL-C levels via the T-cadherin/miR-101-3p/ABCA1 pathway. | 75 | | |
